# Alzheimer’s Disease and Epilepsy: A Perspective on the Opportunities for Overlapping Therapeutic Innovation

**DOI:** 10.1007/s11064-021-03332-y

**Published:** 2021-04-30

**Authors:** Leanne Lehmann, Alexandria Lo, Kevin M. Knox, Melissa Barker-Haliski

**Affiliations:** 1 Undergraduate Neuroscience Program, University of Washington, Seattle, WA 98195 USA; 2 Department of Public Health-Global Health, School of Public Health, University of Washington, Seattle, WA 98195 USA; 3 Department of Pharmacy, School of Pharmacy, University of Washington, Seattle, WA 98195 USA

**Keywords:** Epilepsy, Alzheimer’s disease, Antiseizure drugs, Animal models, Cognitive decline

## Abstract

Early-onset Alzheimer’s disease (AD) is associated with variants in amyloid precursor protein (APP) and presenilin (PSEN) 1 and 2. It is increasingly recognized that patients with AD experience undiagnosed focal seizures. These AD patients with reported seizures may have worsened disease trajectory. Seizures in epilepsy can also lead to cognitive deficits, neuroinflammation, and neurodegeneration. Epilepsy is roughly three times more common in individuals aged 65 and older. Due to the numerous available antiseizure drugs (ASDs), treatment of seizures has been proposed to reduce the burden of AD. More work is needed to establish the functional impact of seizures in AD to determine whether ASDs could be a rational therapeutic strategy. The efficacy of ASDs in aged animals is not routinely studied, despite the fact that the elderly represents the fastest growing demographic with epilepsy. This leaves a particular gap in understanding the discrete pathophysiological overlap between hyperexcitability and aging, and AD more specifically. Most of our preclinical knowledge of hyperexcitability in AD has come from mouse models that overexpress APP. While these studies have been invaluable, other drivers underlie AD, e.g. PSEN2. A diversity of animal models should be more frequently integrated into the study of hyperexcitability in AD, which could be particularly beneficial to identify novel therapies. Specifically, AD-associated risk genes, in particular PSENs, altogether represent underexplored contributors to hyperexcitability. This review assesses the available studies of ASDs administration in clinical AD populations and preclinical studies with AD-associated models and offers a perspective on the opportunities for further therapeutic innovation.

## Introduction

Elderly patients represent the fastest growing demographic with epilepsy, and epilepsy is, in fact, an under recognized comorbidity of Alzheimer’s disease (AD). The relative risk of unprovoked seizures markedly increases in patients with early-onset AD, reaching up to 87-fold greater risk for seizures in individuals with AD onset between 50 and 59 years versus that of the general population [1]. Even late-onset AD patients have a greater incidence of unprovoked seizures relative to that which would be expected in similarly aged individuals (hazard ratio 8.06; 95% confidence interval 3.23–16.61 [2]). Silent hippocampal (focal) seizures have been reported in AD [3]; neuronal hyperexcitability is thus an underexplored contributor to the behavioral sequelae of AD. Epilepsy and AD also share many pathological similarities: temporal lobe atrophy, neuronal death, gliosis, neuritic alterations, and neuroinflammation [4–8]. Both are characterized by neuropsychiatric comorbidities—anxiety, aggression, and depression—that negatively impact quality of life. Both AD and epilepsy are associated with neuronal hyperexcitability; yet how seizures additively or secondarily contribute to the onset and severity of behavioral deficits in AD needs additional study. The purpose of this review is to thus assess the available studies of ASD use for seizures in clinical AD and preclinical studies with AD-associated models, and offer a perspective on the untapped opportunities for further therapeutic innovation.

**Fig. 1:**
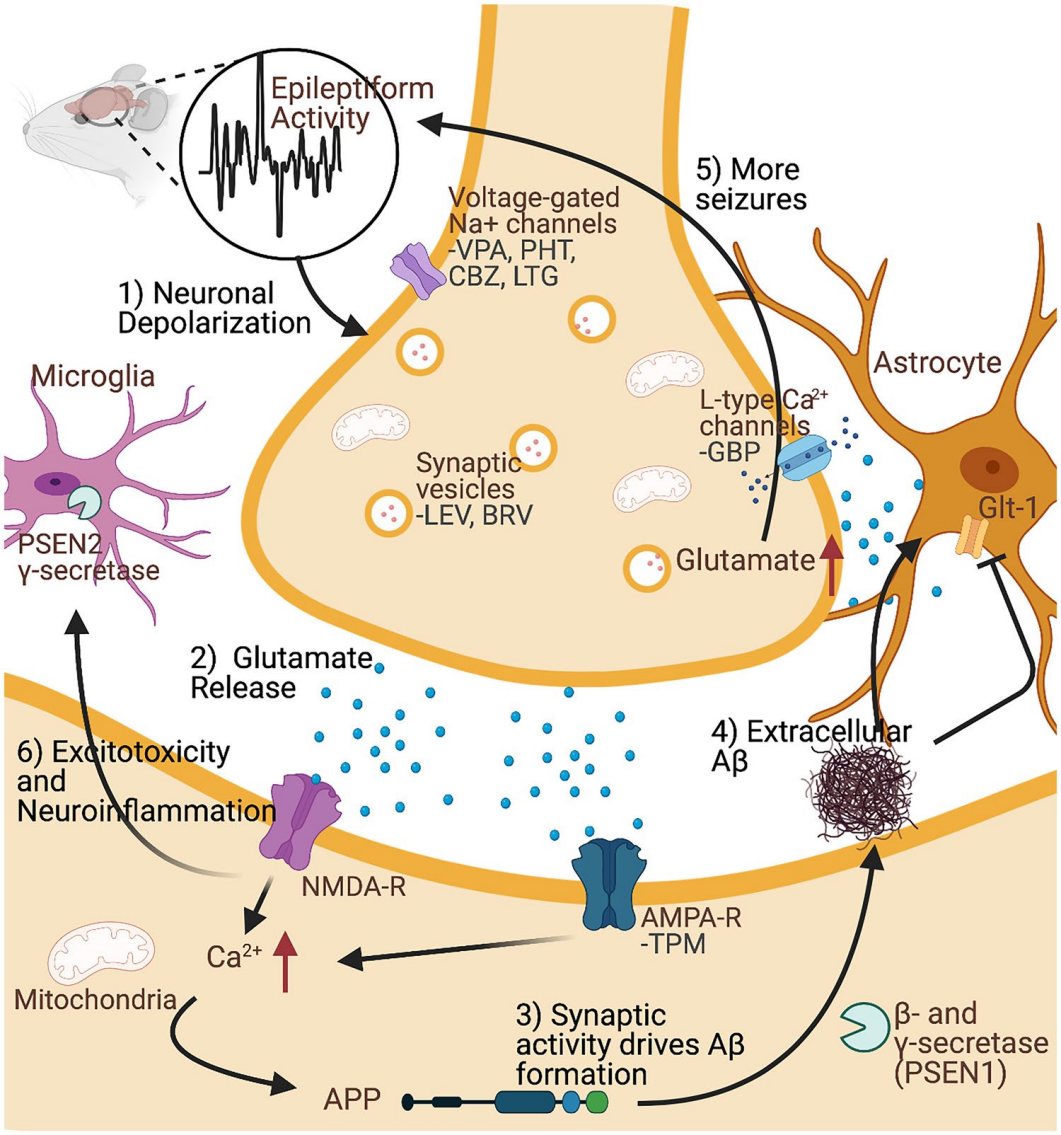
There are numerous aspects of the excitatory synapse that can lead to seizures and disease worsening in Alzheimer’s disease. (1) Through yet undetermined mechanisms, hyperactivity can lead to electrographic seizures that induce neuronal depolarization within hippocampal and cortical brain circuits. Neuronal depolarization leads first to sodium channel opening, and then calcium channel opening. (2) Depolarization induces vesicular trafficking and release of glutamate into the synapse. There, glutamate interacts with both NMDA- and AMPA-type receptors, driving increased intracellular Ca^2+^ levels. (3) High synaptic activity causes the cleavage of amyloid precursor protein (APP) by first β-, and then γ-secretase to generate β- and γ-C-terminal fragments (CTFs) of APP essential to amyloid β (Aβ) plaque formation. (4) Extracellular Aβ induces further glutamate release from astrocytes, as well as blocks the astrocytic Glt-1 transporter that is essential for glutamate reuptake. This effectively increases the amount of glutamate within the neuronal synapse and potentiates NMDA receptor activation, leading to a cycle of neuronal activation and (5) more seizures. This excessive neuronal activity and glutamate-mediated excitotoxicity then further increases neuroinflammation and microglial activation. In the context of Alzheimer’s disease, PSEN2 variants cause dysfunctional microglial response to neuroinflammation and can be proinflammatory, exacerbating neurodegeneration. Several approved antiseizure drugs have been assessed in preclinical AD models and their molecular targets within the excitatory synapse are depicted. This includes agents that act at sodium channels—valproic acid (VPA), carbamazepine (CBZ), phenytoin (PHT), lamotrigine (LTG); those that work at synaptic vesicles—levetiracetam (LEV) and brivaracetam (BRV); those that target Ca^2+^ channels—gabapentin (GBP); and those that act on AMPA-type glutamate receptors—topiramate (TPM). Nonetheless, there are numerous additional antiseizure drugs that work through alternative targets in the excitatory synapse that could be useful to attenuate hyperactivity and excitotoxic neurodegeneration. Created with BioRender.com

The precise mechanisms leading to the development of seizures in the setting of AD are still under investigation and in need of further study. Nonetheless, the mechanisms of seizure generation are well established in the epileptic brain [9]. Neuronal depolarization drives the opening of voltage-gated sodium and calcium channels in the presynaptic neuron, leading to presynaptic release of glutamate into the synaptic cleft (Fig. 1). Post-synaptic α-amino-3-hydroxy-5-methyl-4-isoxazolepropionic acid (AMPA)-type glutamate receptors (AMPARs) are critical to fast excitatory neurotransmission, whereas N-methyl-d-aspartate (NMDA)-type glutamate receptors (NMDARs) mediate much of the slow excitatory potentials essential to global information processing. Glutamate can also interact with ionotropic kainate receptors (KAR), although the precise role of KARs in seizures and neuronal signaling is less clearly established [10]. Presynaptic sodium and calcium channels driving excitatory neurotransmission and post-synaptic glutamate receptors are thus generally relevant therapeutic targets in epilepsy. Further, sodium and calcium channels may contribute not only to seizure generation and maintenance, but also indirectly promote the excitotoxic neurodegeneration of AD through excessive glutamate release [11]. Indeed, the non-competitive NMDAR antagonist, memantine, does provide some degree of benefit in moderate- to severe-AD through the neuroprotective effects of reduced NMDA receptor activation and resulting reductions in the influx of calcium ions [12].

In addition to driving excitotoxic glutamate release, neuronal hyperexcitability may also promote expression of pathological drivers of AD (Fig. 1) causing further neurodegeneration. Synaptic activity drives the cleavage of amyloid precursor protein (APP; [13]) by first β-, then γ-, secretase to form amyloid-β (Aβ) plaques sequestered into the interstitial space [14]. Aβ plaque accumulation is a pathological hallmark of AD. Increased Aβ deposition further indirectly compounds glutamate neurotoxicity via effects on astrocytes (Fig. 1). Under normal conditions, astrocytes remove excess glutamate from the synaptic cleft via the excitatory amino acid transporter 2 (EAAT2/glutamate transporter-1 (Glt-1; [9]). Accumulated Aβ prevents the normal clearance of synaptic glutamate through Glt-1 [15], leading to excessive glutamate levels in the synaptic cleft to the point of extrasynaptic NMDAR activation driving excitotoxic cell death. Aβ also interacts with synaptic NMDARs and enhances calcium influx with neuronal depolarization [16]. Secondarily, Aβ plaque accumulation may lead to the misfolding of tau protein and aggregation, a neuronal microtubule-associated protein that plays a core role in axonal transport. Tau protein is hyperphosphorylated in AD, disrupting normal neuronal functions. Accumulation of hyperphosphorylated tau protein leads to the neurofibrillary tangles that are a defining feature of clinical AD [17, 18]. Accumulation of neurofibrillary tangles can cause neurodegeneration and cognitive deficit. Thus, neuronal hyperactivity leads to a number of pathological changes in the AD brain that additively increase seizure susceptibility, promote excitotoxic neuronal death, and Aβ accumulation (Fig. 1).

**Fig. 2:**
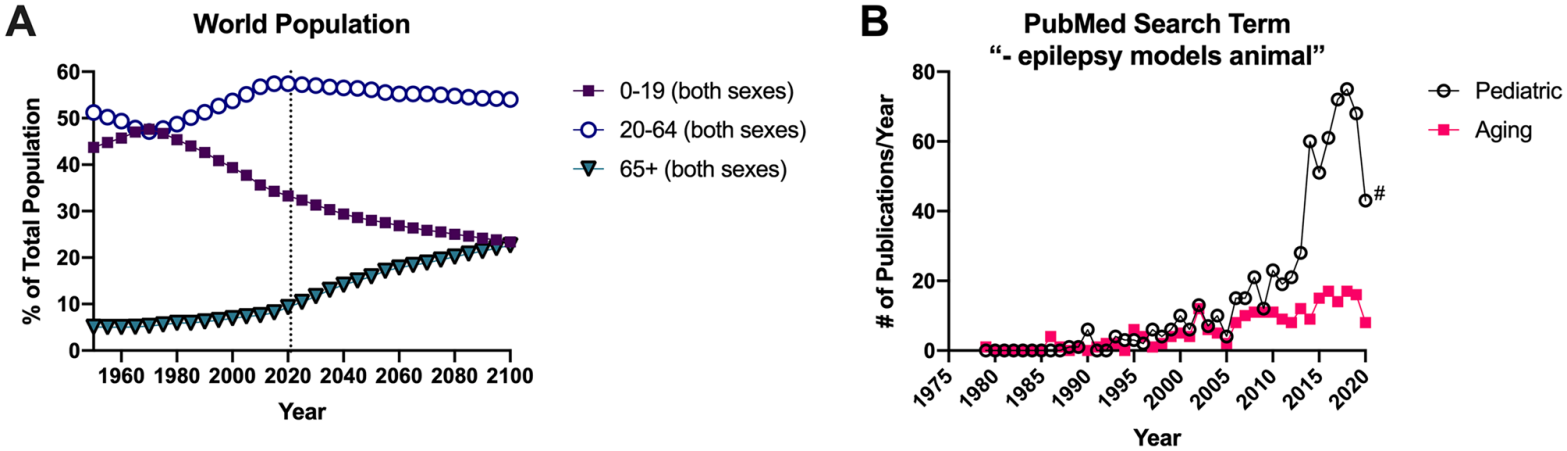
**a** The percentage of people aged 65+ has steadily increased as a total percentage of the global population since 1950. Children aged 0–19 represent a progressively smaller proportion of the global population whereas elderly people aged 65  increasingly make up a larger proportion of the worldwide population. Elderly patients represent the fastest growing demographic with epilepsy, likely as a result of the numerous inciting events that can cause epilepsy in the elderly (e.g., stroke, brain tumor, traumatic brain injury/falls). Dashed line at 2020 represents estimated populations going forward from UN Population database access date of December 14, 2020 (https://population.un.org/wpp/DataQuery/). **b** In the preclinical space, there has been relative concordance between the publications with aged and pediatric animal models until approximately 2013–2014, likely as a result of several research initiatives (e.g., Citizens United for Research in Epilepsy Infantile Spasms Initiative, 2013 NINDS Curing the Epilepsies Meeting, etc.). Since this time, published studies using pediatric epilepsy models have significantly accelerated whereas similar publications with aged animal models of epilepsy has not similarly increased. This represents a significant gap in preclinical research that does not match the clinical patient demographic needs. # indicates reporting of all studies published up until the Pubmed access date of December 14, 2020

Seizure control with antiseizure drugs (ASDs) may be a potential strategy to reduce the burden of AD [19]. With over 30 FDA-approved ASDs [20], there is an untapped opportunity to repurpose ASDs to possibly curb the severity of AD. Indeed, efforts have been made in recent years to assess the potential disease-modifying effects of ASD administration in AD, including findings that levetiracetam (LEV) may be disease-modifying [21–23]. However, insufficient clinical or preclinical studies have been conducted to definitively establish whether seizures in AD patients or animal models are not also sensitive to other ASDs. Moreover, the clinical studies that have been performed have not been uniformly conducted such that direct comparisons across studies is quite challenging. A considerable proportion of patients with epilepsy do not receive therapeutic benefit from available ASDs [24]. In this regard, preclinical models of AD are essential to simultaneously explore in greater detail the mechanisms associated with seizures in AD and potentially uncover novel treatments for hyperexcitability, not to mention rigorously establish the efficacy of ASDs in an aged AD-associated neurological substrate. The worldwide percentage of people aged over 65 is increasing considerably (Fig. 2a), but basic science has not sufficiently responded to address this clinical need. ASD discovery is not traditionally conducted in aged rodents [25–28]. Just as the use of juvenile *Scn1a* ± mice has uncovered novel treatments for Dravet syndrome (e.g., cannabidiol [29]), a rare pediatric epileptic encephalopathy, there is substantial opportunity to more frequently integrate aged animals, and in particular aged AD models, into ASD discovery to advance novel treatments and identify novel therapeutic targets (Fig. 2b). Clinical and preclinical evidence reveals a chance to prioritize ASD discovery for seizures in AD. Such efforts may also translate to improved treatments for epilepsy, in general. Seizures could be a manageable feature of AD [30–32] because seizures in the elderly are generally not drug-resistant [33]. However, it is essential to *clearly establish the direct contribution* of seizures on AD burden and neuropathology so that future preclinical and clinical trials can be more rationally and rigorously conducted.

## Clinical Efficacy of Antiseizure Drugs in Patients with Alzheimer’s Disease

The focal seizures in AD may be difficult to recognize [3, 31] and may even go undetected by surface EEG electrodes that only detect cortical activity [3]. Undetected seizures are untreated seizures. Whether the onset or severity of behavioral sequelae of AD is exacerbated by uncontrolled focal seizures is presently unclear. Synaptic activity itself can drive the release of Aβ [14]. It is therefore reasonable to presume that administration of ASDs could reduce the accumulation of Aβ through the direct suppression of seizures and hyperexcitability (Fig. 1).

Limited studies have suggested that pharmacological control of focal seizures with ASDs administered to patients with AD may slow disease progression and reduce the severity of neuropsychiatric comorbidities [22]. Yet, the evidence that ASDs have therapeutic potential in clinical AD has been mixed (Table 1, [19]). Furthermore, there has been no standardized approach to patient enrollment, ASD selection, or trial methodology to rigorously assess the therapeutic potential of ASD administration in a homogenously defined AD patient population. Limited clinical evidence would suggest that ASDs carry the potential to improve cognitive function in mild cognitive impairment [21], highlighting an untapped opportunity to improve clinical management of AD. For example, in a randomized study, valproic acid (VPA; 10–12 mg/kg/day) or placebo was administered to 313 participants with moderate AD over 24 months (122 patients completed the study), yet the study concluded that VPA was associated with brain volume loss [34]. In another randomized study, 313 individuals with mild-to-moderate AD were given VPA (10–12 mg/kg/day) or placebo for a 24-month period and again demonstrated a greater loss of hippocampal and brain volume in the VPA treatment group [35]. Furthermore, VPA administration led to adverse effects and no reduction in disease burden. VPA exerts a broad efficacy profile in patients with epilepsy and the precise mechanism by which it exerts an anticonvulsant effect is still unclear [20]; whether a specific molecular target could be more useful for the seizures in AD is currently unknown. Available evidence thus-far clearly does not indicate that all ASDs are the panacea for AD and that inappropriate ASD selection may, in fact, carry the potential to do further harm.

**Table 1:** Clinical studies of ASD efficacy and tolerability in patients with Alzheimer’s disease

Clinical studies				
Antiseizure Drug (abbreviation)	Clinical trial design and patient demographics (if stated)	Beneficial (Positive) effects	Adverse (Negative) effects	Reference(s)
Valproic Acid (VPA; 10–12 mg/kg/day)	24 month randomized, double-blind placebo-controlled Mild to moderate AD		VPA-treatment group had increased rates of brain volume loss During the first 12 months, the VPA-treatment group had an accelerated decline in MMSE scores	[35]
	Randomized, double-blind placebo-controlled Moderate AD		VPA-treatment group had a greater mean brain volume loss At 12 months, the VPA-treatment group had more rapid decline of MMSE scores VPA administration was associated with adverse effects (e.g. somnolence, asthenia, tremors)	[34]
Levetiracetam (LEV; 1000–1500 mg/day)	Observational study AD patients (n = 25) determined with CT/MRI and diagnosed with epileptic seizures	72% of patients were seizure-free at follow-up during the 14–25 month period	LEV administration was associated with adverse effects; e.g. somnolence and gait abnormality in 4/25 patients	[36]
	Randomized, three-arm parallel-treatment group, case–control, AD patients with seizures (n = 95): LEV (n = 38), PB (n = 28), LTG (n = 29) 4-week dose adjustment and a 12-month evaluation period	MMSE scores (+ 0.23) and ADAS-Cog (0.23) scores showed improvement Associated with improved oral fluency, short-term memory, and attention 29% (11/38) became seizure free 71% responder rate after 12 months	Reported central nervous system-related and mild adverse effects (e.g., dizziness, headache, asthenia, and somnolence) None of the adverse effects required discontinuation of treatment Not statistically significant adverse events from PB and LTG groups Mood score worsened (+ 0.20 on Cornell scale)	[22]
Lamotrigine (LTG; range of 25–100 mg/day)	Randomized, three-arm parallel-treatment group, case–control, AD patients with seizures (n = 95): LEV (n = 38), PB (n = 28), LTG (n = 29) 4-week dose adjustment and a 12-month evaluation period	Generally better scores on measurements of mood (− 0.72 on the Cornell scale) 59% responder rate at 12 months	28% (7 patients) reported mild adverse effects: somnolence, dizziness, headache None of the patients withdrew due to side effects Not statistically significant adverse events from LEV and PB groups Slight declines in MMSE (-0.64) and ADAS-Cog (+ 0.680)	[22]
Phenobarbital (PB; range of 50–100 mg/day)	Randomized, three-arm parallel-treatment group, case–control, AD patients with seizures (n = 95): LEV (n = 38), PB (n = 28), LTG (n = 29) 4-week dose adjustment and a 12-month evaluation period	64% responder rate after 12 months	Not statistically significant adverse events from LEV and LTG groups Lower mean scores indicate a significant worsening of cognitive performance at 6 and 12 months on MMSE (− 1.57) and ADAS-Cog (+ 0.174) Mood score worsened (+ 1.74 on Cornell scale) 17% withdrawal rate 43% experienced adverse effects (e.g., somnolence and asthenia) 61% experienced side effects	[22]

*ADAS-Cog* Alzheimer's Disease Assessment Scale-Cognitive Subscale Scoring system, *MMSE* mini-mental state exam

Rational selection of ASDs based on specific mechanism may instead be more beneficial in AD (Table 1). For example, LEV monotherapy (1000–1500 mg/day) in 25 patients with advanced AD and seizures demonstrated that 72% of patients remained seizure-free over 14–25 months, suggesting that LEV could improve seizure control in AD [36]. A different randomized prospective study of 95 AD patients aged 60–90 years old with documented seizures was designed to compare the anticonvulsant efficacy of LEV (n = 38), phenobarbital (PB; n = 28), and lamotrigine (LTG; n = 29); there were no detectable differences in the 12-month change in seizure freedom and responder rate [22]. As a secondary objective, that study also assessed the cognitive impact of ASD administration to AD patients with seizures versus the cognitive performance of AD patients without seizures [22]. PB evoked negative cognitive effects, LEV improved attention, short-term memory, and oral fluency, and LTG conferred better moods [22]. Sodium channel-blocking ASDs, like LTG, have gained interest and off-label use for the management of impulsive aggression [37–39], suggesting that rationally selected ASDs may attenuate specific AD-associated neuropsychiatric symptoms. Pharmacological control of undetected focal seizures in AD with appropriately and rationally selected ASDs (e.g., LTG for neuropsychiatric deficits vs LEV for cognition) could potentially improve quality of life and prolong the period of independent function. LEV was developed as an enantiomer of the nootropic agent, piracetam [40], so studies to further elucidate the cognitive enhancing effects of LEV and related compounds (e.g. brivaracetam) in AD are warranted. However, whether LEV and LTG, or other ASD combinations, could additively or synergistically reduce the burden of comorbidities of AD must be first rigorously established in well-controlled clinical trials. Studies in AD patients should also be prioritized to assess the antiseizure potential of newer ASDs (e.g. cannabidiol) that have been found to benefit cognitive function in patients with epilepsy [41] or animal epilepsy models [42].

ASD use in the elderly is already high; at least 10% of nursing home residents take at least one ASD [43–45]. Older adults with chronic conditions pose a particular prescribing challenge due to the high potential for drug-drug interactions [46], which can limit medication adherence due to adverse drug effects and/or adversely affect disease outcomes. Careful selection of ASDs in elderly populations is particularly important because many of the available ASDs can alter the metabolism and/or bioavailability of drugs that are also prescribed for other aging-related conditions [47]. For example, in a random sample of 5% of Medicare beneficiaries collected from 2008 to 2010, roughly 1 in 4 incident cases of epilepsy received an ASD in combination with at least one non-ASD that could adversely affect pharmacokinetic interactions between the agents [46]. In particular, over 50% of older adults with epilepsy continue to use phenytoin (PHT) after 12 months [48], yet PHT is associated with high potential for drug-drug interactions in this population [46]. Moreover, poor adherence to specific medications by older adults can also put individuals at a higher risk of dementia [49]. Elderly patients with seizures are thus a particularly challenging group for drug treatment due to the numerous potential risks associated with polypharmacy. Selection of ASDs in combination with other medications used in this patient population should be carefully and rigorously assessed with both predictive animal models and carefully designed clinical studies, prior to widespread clinical implementation in patients with or at risk for AD.

## Preclinical Models of Aging and Epilepsy Should Address Clinical Needs

Despite the greater incidence of epilepsy in elderly individuals and increased risk of comorbid seizures in AD patients, few aged animal seizure and epilepsy models have been sufficiently characterized to support drug discovery for aging-related seizures [50]. Comparative pharmacology with prototypical ASDs has not been extensively collected in the available animal models of AD to inform on the potential pharmacokinetic, toxicity, or drug interactions in aged or geriatric populations. This is in stark contrast to the rigorous evaluation of comparative pharmacology in rodent drug-resistant epilepsy models [26, 51–53]. Information concerning the risk of seizure in rodents with AD-associated mutations would support their utility for moderate- to high-throughput preclinical approaches for drug development for aged and geriatric patients with seizures. Mouse strain alone can significantly impact seizure threshold [54], as can age [55], but whether there are additive effects of aging and AD-associated mutations on seizure susceptibility or ASD efficacy should be more comprehensively assessed.

Preclinical models are invaluable to predict tolerability and pharmacokinetics in a specific population, e.g. aged individuals. However, the ASDs on the market were brought forth based on efficacy in young adult, neurologically intact wild-type rodents [56]. Preclinical information concerning the efficacy and safety of investigational agents is not routinely defined in aged animals with seizures [28, 56]. While there has been a recent explosion in available preclinical models of pediatric epilepsy used for ASD discovery (Fig. 2b), there has not been a similar rise in the use of aged animals. Rodents with AD-associated variants are simply not used for ASD identification or differentiation [57]. Safety and tolerability of FDA-approved ASDs in aged individuals has been established in clinical trials [58–60], even though aged rodents share many of the age-related physiological changes of humans [61] and could be important surrogates to predict tolerability for this demographic. For example, we detect marked tremors in aged mice acutely treated with high doses of LTG [62], an adverse effect also reported in elderly patients treated with LTG [63]. It is plausible that use of aged rodents for ASD tolerability testing could have predicted such observations, as has already been done with other rodent epilepsy models [64, 65]. As a result, the management of seizures in elderly patients, including those with seizures in AD, may be underinformed.

Preclinical studies have defined the cognitive profile, baseline EEG activity, and changes in synaptic morphology associated with APP overexpression at a single age in mice [66–68], yet the basic understanding of age-related susceptibility to seizures in the presence of AD-associated risk genes is still relatively undefined. Rodent models with early onset-AD-associated variants are particularly valuable to define how hyperexcitability may be associated with AD throughout life. While the age of AD onset varies markedly based on the specific genetic variant, the pathology and clinical course of early-onset AD is similar to that of sporadic AD [69]. Early-onset AD is associated with point mutations or indels within PSEN1, PSEN2 or APP genes, or with a duplication of APP. However, the majority of our preclinical knowledge of hyperexcitability in AD has come from mice that overexpress APP [23, 67]. While spontaneous seizures are well-tolerated in most APP/PS1 mice, ~ 38% of animals can succumb to seizure-related mortality [68], constraining resources and driving up costs for moderate-throughput ASD discovery [57]. Thus, models that overexpress early-onset AD-associated APP variants have been helpful to explore the manifestation of hyperexcitability in AD, but there still remains a need to establish whether and how other drivers of AD affect seizures and disease course.

There is scattered preclinical data assessing the impact of ASDs on disease burden in a diversity of AD models; that which is available also predominantly focuses on the effects of ASDs on behaviors in models that overexpress APP as a result of early-onset AD-associated genetic variants. Furthermore, studies conducted thus far do not consistently nor uniformly apply anticonvulsant doses of ASDs to discretely interrogate antiseizure versus disease-modifying effects (Table 2). For example, chronic (4-week) administration of low dose VPA to 7- to 9-month-old APP23 transgenic mice reduced neuritic plaques (i.e. amyloid plaques) by 56–76% and improved spatial memory. A similar course of VPA administered to 6-week-old APP23/PS45 double-transgenic mice (which express presenilin-1 with G384A variant) resulted in a nearly 80% reduction in plaques [70]. However, the dose of VPA (30 mg/kg, i.p., q.i.d.) used in either strain was far below that which acutely blocks focal seizures in wild-type C57Bl/6 mice (169–276 mg/kg, i.p.) [51], including similarly aged C57Bl/6 mice (186 mg/kg, i.p.) [62]. The frequency of administration (once/day) was also insufficient to attain steady-state seizure suppression with such a rapidly metabolized ASD in mice [71]. Thus, the effects of once-daily VPA administration on Aβ plaques in APP23 and APP23/PS45 mice was likely not due to direct seizure control [72, 73]. Repeated once-daily i.p. administration of topiramate (20 mg/kg), LEV (50 mg/kg), or VPA (30 mg/kg) to 7-month-old APP/PS1 mice reduced Aβ plaques by 45.8%, 51.9%, and 53.1% respectively, and attenuated behavioral deficits [74]. However, as with the studies by Qing and colleagues [70], the doses and frequency of topiramate and LEV administration of [74] were well below the acutely anticonvulsant doses in mice [71], suggesting that any disease-modification was not directly attributable to prevention of electrographic seizures.

**Table 2:** Preclinical studies of ASD efficacy and tolerability on acute or chronic seizures (evoked or spontaneous) in animal models of Alzheimer’s disease-associated genetic variants

Preclinical studies				
Antiseizure drug (abbreviation)	AD Mouse Model	Beneficial (Positive) effects	Adverse (Negative) effects	References (including Dose, Route, and Frequency of ASD Administration)
Valproic Acid (VPA)	APPswe/PS1dE9 [68, 74, 75, 127] hAPPJ20 [23] APP23/PS45 [70] ICV-STZ induced mouse model of SAD [125]	Decreased neuritic plaque [70, 74, 127] Reversed behavioral deficits [74] Upregulated Aβ transport across the BBB [74] Reduced tau phosphorylation [74] Improved memory deficits [70] Reduced epileptiform discharges [68] Improved cognitive functions [125] Increased neprilysin levels [125] Increased ACh levels and decrease AChE activity [125] Relieved manic/anxiety symptoms of male mice [127] Prevented loss of synapses in the hippocampus [127] Prevented neuronal cell death [127]	Did not reduce spike frequency [23] Not effective in reducing agitation or aggression [70] Reduced effect of epileptiform activity is not long-lasting after treatment is discontinued [75] Significant improvements in learning and memory only seen in male mice, not females [127]	[74]—30 mg/kg, i.p. q.i.d. for 30 days [23]—single dose of 300 mg/kg, i.p. for electrographic seizure studies [70]—30 mg/kg, i.p. q.i.d. for 4 weeks [68]—260 mg/kg, i.p. b.i.d for 3 days or 400 mg/kg, i.p. b.i.d for 5 days for electrographic seizures studies [75]—30 or 300 mg/kg, i.p. q.i.d for 7 days for electrographic seizure studies [125]—100 or 200 mg/kg/day i.p. for 21 days [127]—30 mg/kg/day i.p. for 4 weeks
Topiramate (TPM)	APPswe/PS1dE9 [74, 126]	Decreased neuritic plaque [74, 126] Reversed behavioral deficits [74] Upregulated Aβ transport across the BBB [74] Reduced tau phosphorylation [74] Increased frequency of interactive behavior and nest building activity [126]		[74]—20 mg/kg, i.p. q.i.d. for 30 days [126]—20 mg/kg, p.o. for 21 days
Levetiracetam (LEV)	APPswe/PS1dE9 [72, 74] hAPPJ20 [23] 3xTg-AD [128] 5xFAD [129]	Decreased neuritic plaque [74] Reversed behavioral deficits [23, 74] Upregulated Aβ transport across the BBB [74] Reduced tau phosphorylation [74] Effective in reducing spike/seizure-indicating spike frequency [23, 72] Improved learning and memory [23] Reversed synaptic deficits in hippocampus [23] Reversed the frequency-dependent reduced firing rates of cortical pyramidal cells and promoted uncoupling of inhibitory interneurons and pyramidal cells in cortex [128]	Did not reduce levels of hAPP or Aβ [23] Loss of antiepileptic effect at high doses [23] Not able to rescue memory deficits [129]	[72]—single dose of 75 mg/kg, i.p. 30 min prior to electrographic seizure studies [73]—single dose of 20 mg/kg, i.p. q.i.d. once for electrographic seizure studies [74]—50 mg/kg, i.p. q.i.d. for 30 days [23]—200 mg/kg, i.p. q.i.d. for 30 days [128]—single dose of 200 mg/kg i.p. 2 hours prior to electrographic seizure recordings [129]—single dose of either 10 or 20 mg/kg, i.p
Ethosuximide (ESM)	APPswe/PS1dE9 [72, 73] hAPPJ20 [23]	Reduced spike wave discharges [73]	Not effective in reducing spike/seizure-indicating spike frequency [23, 72] Not effective in reversing memory impairments [23] Did not change levels of Aβ or plaques in the brain [23]	[72]—single dose of 200 mg/kg, i.p. 30 min prior to electrographic seizure studies [73]—200 mg/kg, i.p. q.i.d. once for electrographic seizure studies; 30 mg/mL in drinking water for 30 days for disease-modification studies [23]—single dose of 400 mg/kg, i.p. prior to electrographic seizure studies
Brivaracetam (BRV)	APPswe/PS1dE9 [73]	Reduced spike wave discharges [73] Fully reversed memory impairments [73]	Did not change levels of Aβ or plaques in the brain [73]	[73]—single dose of 10 mg/kg, i.p. for electrographic seizure studies; 8.5 mg/kg/day in via i.p. osmotic pump 280 days for disease-modification studies
Phenytoin (PHT)	3xTg-AD [73] hAPPJ20 [23] APPswe/PS1dE9 [68]	Reduced epileptiform discharges [68]	No effect on spike-wave discharges [73] Increased electrographic spike frequency by > 183% of baseline[23]	[73]—single dose of 20 mg/kg, i.p. for electrographic seizure studies [23]—single dose of 100 mg/kg, i.p. for electrographic seizure studies [68]—10–40 mg/kg, i.p. t.i.d for 3 days for electrographic seizure studies
Gabapentin (GBP)	hAPPJ20 [23]		Did not change electrographic spike frequency from baseline [23]	[23]—single dose of 100 mg/kg, i.p. for electrographic seizure studies
Pregabalin	hAPPJ20 [23]		Increased electrographic spike frequency by > 87% of baseline [23]	[23]—single dose of 200 mg/kg, i.p. for electrographic seizure studies
Vigabatrin	hAPPJ20 [23]		Did not change electrographic spike frequency from baseline [23]	[23]—single dose of 300 mg/kg, i.p. for electrographic seizure studies
Carbamazepine (CBZ)	APPswe/PS1dE9 [68] 3xTg-AD [130]	Reducedd epileptiform discharges [68] Improved spatial learning ability [130] Reduced amount of Aβ plaques in the hippocampus [130] Upregulated LC3-II to stimulate autophagy in the hippocampus (independent of mTOR pathway) [130]	Dosage had toxic effects [130]	[68]—10–40 mg/kg, i.p. t.i.d for 5 days for electrographic seizures studies [130]—100 mg/kg/day p.o. for 2 months
Diazepam (DZP)	OSK-KI APP [131] APP Tg2576 [132]	Improved memory [131] Prevented Aβ oligomer accumulation and synapse loss [131] Reduced Aβ deposits in CA1, frontal cortex, and entorhinal cortex [132]		[131]—2 μg p.o. 5x/week for 2 months [132]—4 mg/kg/week for 4 weeks
Lamotrigine (LTG)	APP/PS1 [133, 134]	Reduced expression of IL-6 and IL-1β in brain tissue^1^ Suppressed GFAP overexpression in the brain, indicating an inhibitory effect on astrocytes [133] Prevented executive dysfunction and long-term memory impairment [133] Enhanced learning and memory [133] Prevented impairment in synaptic plasticity [134] Prevented neuronal loss [134] Inhibited the generation of Aβ leading to reduction in density of amyloid plaques [134] Suppressed activation of microglia and astrocytes [134] Reduced epileptic spikes in the cortex and hippocampus [134] Enhanced levels of BDNF and NGF in the brain [134]	Not able to rescue short-term memory deterioration [133]	[133]—30 mg/kg/day p.o. for 6 months [134]—30 mg/kg/day p.o. for 2–5 months

Studies that have directly assessed the antiseizure effects of ASDs in preclinical models with AD-associated genotypes are even more limited (Table 2). These studies have consistently relied on APP overexpressing mouse models. The doses that were assessed in such studies were not also uniformly applied to encompass an anticonvulsant range. Ziyatdinova and colleagues demonstrated a beneficial dose-related effect of VPA (30 vs 300 mg/kg, i.p.) on spontaneous electrographic discharges (EDs) in APP/PS1 mice [75]. Yet, there was no associated effect of VPA (300 mg/kg) on soluble and insoluble Aβ levels in that study [75]. Low-dose LEV (20–75 mg/kg) has been found to consistently suppress spontaneous EDs in APP/PS1 [72, 73] and hAPPJ20 mice [23]. This dose range of LEV is anticonvulsant against focal seizures in young and aged C57Bl/6 mice [51, 62]. Acute administration of the T-type calcium channel blocker, ethosuximide (ESM; 200 mg/kg), can also reduce EDs in APP/PS1 mice [72, 73] but high dose ESM (400 mg/kg) is ineffective in hAPPJ20 mice [23]. This dose of ESM is also acutely anticonvulsant in a mouse audiogenic seizure model of reflex seizure [71] and myoclonic seizures [76]. Brivaracetam (8.5 mg/kg/day for 28 days) has been shown to not only reduce EDs, but reverse memory impairments in APP/PS1 mice [73], likely due to anticonvulsant effects on EDs. Notably, brivaracetam has a superior profile of brain bioavailability in rodents, more rapid penetration into the brain, and is substantially more potent than LEV in numerous acute seizure models in male mice, including this 8.5 mg/kg/day dose [77, 78]. Certainly, some specific ASDs have been found to effectively suppress seizures in mouse AD models.

The spontaneous seizures of APP/PS1 mice aged 4- to 6-months-old were also found to be sensitive to repeated administration of anticonvulsant doses of the sodium channel-blocking ASDs, carbamazepine (CBZ) and phenytoin (PHT), and the broad spectrum ASD, VPA [68]. However, that study also demonstrated that high dose administration of the sodium channel-blocking ASDs (40 mg/kg CBZ; 40 mg/kg PHT) worsened the electrographic discharges of 1–3 individual mice [68], consistent with later findings with similarly high dose of PHT (40 mg/kg) in hAPPJ20 mice [23]. While these findings for seizure worsening with high dose administration of sodium channel blocking ASDs is at first glance concerning for a mouse model of AD, it is well established that sodium channel blockers can lower seizure threshold at high doses [76, 79], thus care should be taken to not over interpret these findings as specific to AD models. Lastly, Jin and colleagues have also more recently demonstrated an effect of ESM (200 mg/kg) and LEV (75 mg/kg) on state-dependent spike-wave discharges (SWDs) in aged APP/PS1 mice [80]. This study highlights the capacity to evaluate the efficacy of ASDs on specific types of epileptiform activity that is also observed in genetically susceptible rat models of absence epilepsy [57, 81, 82]. There is scattered evidence that ASDs can effectively reduce spontaneous EDs and SWDs in mice that overexpress APP. However, the studies have not been uniformly conducted using well-established principles for pharmacological studies [51], and the outcome measures have been mixed, clouding interpretation of any findings and limiting translational impact. Additional studies to comprehensively assess whether anticonvulsant doses of other ASDs affect seizures in other AD-associated models that do not exclusively overexpress APP are also needed.

## Overlapping Molecular Targets in Epilepsy and Alzheimer’s Disease

In addition to the shared pathophysiology between seizures and AD, which can potentially worsen functional outcomes, these disorders share several similarities in molecular drivers of disease that warrant further study. The formation of Aβ plaques as a result of pathological neuronal hyperactivity and cleavage of APP by first β-, and then γ-secretase to generate β- and γ-C-terminal fragments (CTFs) of APP is essential to Aβ plaque formation produces a key clinical feature of AD (Fig. 1). However, the α-secretase A Disintegrin And Metalloprotease 10 (ADAM10) is involved in a secondary and understudied pathway of APP processing that releases the soluble portion (sAβPPα) and prevents the formation of senile plaques [83]. In this manner, ADAM10 may actually be protective in AD and prevent the formation of senile plaques. Interestingly, ADAM10 has been implicated in the pathogenesis of focal cortical dysplasia [84, 85], a disease characterized by epileptic seizures and neurocognitive deficit. Overexpression of ADAM10 in a mouse temporal lobe epilepsy model also attenuates the burden of seizures and prevents pathological neuroinflammation [86]. While no ASD has yet been shown to affect the ADAM10 pathway, this work highlights that this alternative pathway underlying pathogenesis in both epilepsy and AD is a potential avenue for therapeutic intervention.

Both epilepsy and AD are increasingly viewed as disorders associated with significant metabolic dysfunction. Glucose hypometabolism is commonly observed in both clinical [87] and preclinical studies [88] of AD, as is the compensatory shift to alternative fuel sources, e.g. ketone bodies [89–91]. Reduced glucose utilization in the hippocampus and entorhinal cortex, two brain regions also heavily implicated in temporal lobe epilpesy, correlates to cognitive deficits over time in normal aged individuals, and can also predict those individuals who go on to develop mild cognitive impairment [92]. Oxidative stress is often reported in preclinical models of temporal lobe epilepsy in adult rodents [93]. Moreover, the aged brain itself may also undergo significant shifts in normal bioenergetics processes; aged individuals utilize glucose to alternative fuel sources (e.g. ketone bodies) at a ratio of 29:1, whereas young individuals exclusively utilize glucose at a ratio of 100:0 [94]. In patients with AD, this ratio of glucose to alternative fuel consumption is 2:1 [94]. Thus, age-related shifts in glucose metabolism may contribute to both epilepsy and AD. Further, reducing glycolysis in both patients with epilepsy and animal models using 2-deoxy-D-glucose (2DG) has been found to be quite effective as an anticonvulsant strategy [95, 96]. Thus, metabolic regulation through pharmacological or dietary manipulation is an overlapping therapeutic target for both epilepsy and AD, as well as potentially affecting the hyperexcitability associated with seizures in patients with AD.

## Presenilins are an Underexplored Molecular Contributor to Seizures in AD

Early-onset AD is also associated with PSEN1 and PSEN2 variants [97]. Presenilins are intramembrane proteases of the catalytic component of γ-secretase; mutations of which promote the formation of cleavage products such as Aβ (Fig. 1; [69, 98]). There is high incidence of seizures (32%) in AD patients with the most common PSEN2 variant (N141I), although these events are only self-reported and no chronic monitoring for electrographic (focal) seizures has yet been exclusively conducted in this patient group [99]. Moreover, similar to case reports in other patients with early-onset AD [3, 19, 32], it is entirely plausible that focal seizures are more frequent with PSEN2 variants than generalized seizures that would be readily detected by a caregiver, underscoring the need for more detailed clinical monitoring. PSEN2 is also particularly intriguing to explore the hyperexcitability of AD because some PSEN2 variants are associated with reduced penetrance [100], such that cases may be inadvertently masked as sporadic AD [101].

PSEN2 is attractive to define the additive impact of aging and seizures on disease burden in AD. First, PSENs may more meaningfully influence neuropsychiatric symptoms of AD [102]. Second, PSEN2 is a key contributor underlying neuroinflammation [103, 104]; loss of normal PSEN2 function disrupts canonical γ-secretase activity to promote a pro-inflammatory phenotype mediated by microglial activation and cytokine release [104, 105]. Microglial activation and cytokine release can also promote the development of epilepsy [106, 107]. Use of PSEN2 transgenic versus APP overexpression models [67, 108] allows for the interrogation of the role that an altered neuroinflammatory milieu may play on seizure susceptibility and burden of AD. Third, PSEN2 variants cause AD with later onset than PSEN1 variants (AD Mutation database: https://www.alzforum.org/mutations), making PSEN2 variant models suitable to simultaneously interrogate the additive environmental impacts of senescence [109] and neuronal hyperexcitability on disease outcomes. Fourth, PSEN2 variants do not induce Aβ accumulation in mice [110] and APP processing [111], unlike APP/PS1 transgenic mouse models [112, 113], affording an opportunity to define whether Aβ accumulation and/or seizures are more detrimental to the onset and severity of behavioral sequelae. Finally, PSEN2 is essential to mitochondrial-dependent Ca^2+^ homeostasis underlying normal neuronal signaling [114, 115]. Specifically, PSEN2, but not PSEN1, regulates the tethering and Ca^2+^ crosstalk between the endoplasmic reticulum and mitochondria, which may itself alter cellular bioenergetics or increase mitochondria-dependent cell death [115]. The early-onset AD-associated PSEN2-N141I variant, but not normal PSEN2 or PSEN1, can potentiate mitochondrial dysfunction [116]. Neuronal hyperexcitability due to dysregulation of Ca^2+^ release may be one of the first observable biomarkers in the aged and diseased brain and is consistently observed in rodent models of AD [117–119] and epilepsy [120]. Altogether, PSEN2 mouse models should be increasingly integrated into studies of seizures and hyperexcitability in AD to interrogate pathological processes of AD that are distinct from extraneous Aβ production.

Mouse PSEN2 variant models are an untapped opportunity to further elucidate the mechanism of seizures in AD. It is clear that APP overexpression in mice has revealed significant pathophysiological overlap between AD and epilepsy [67, 121]; yet few studies have extensively evaluated whether other AD risk genes (e.g., PSENs) are similarly associated with changes in seizure susceptibility, seizure-induced functional impacts, or ASD efficacy. Recently, we have begun to explore age-related seizure susceptibility in mice with loss of normal PSEN2 function. We observe an age-related change in the development of kindled seizures, a model of epileptogenesis [81], in PSEN2 knockout (KO) mice [62]. PSEN2 variants in AD lead to a loss of normal γ-secretase activity, such that PSEN2 KO mice are useful to a priori assess how loss of normal PSEN2 function influences seizure susceptibility. Young PSEN2 KO mice were less susceptible to formation of an epileptic network than aged PSEN2 KO mice [62], implicating an age-related change in neuronal excitability and susceptibility to chronic seizures with loss of normal PSEN2 function. The latency to develop corneal kindled seizures in 2-month-old PSEN2 KO mice was significantly longer than age- and sex-matched WT mice; an effect that was lost in mice aged > 8 months [62]. Notably, the seizure duration of mice with fully kindled seizures was significantly longer in young PSEN2 KO mice, despite requiring more corneal stimulations to achieve the fully kindled state. The seizures of young male fully kindled PSEN2 KO mice were also less sensitive to escalating doses of LEV and diazepam (DZP) than age-matched WT mice, as well as the fully kindled PSEN2 KO mice aged greater than 8-months old. Thus, corneal kindling of PSEN2 KO mice is a technically feasible way to assess the potential for anticonvulsant efficacy, identify novel contributors to ictogenesis, and assess age-related changes in seizure susceptibility. In addition to assessing kindling acquisition rates of young and mature PSEN2 KO mice, we assessed the age-related changes in seizure susceptibility using two acute seizure tests: the minimal clonic and 6 Hz seizure models [62]. Importantly, these studies established the feasibility of using well-established, moderate-throughput acute and chronic seizure models [26, 57] to PSEN2 KO mice to define how AD-associated risk factors impact ASD efficacy and seizure susceptibility. PSEN2 variant models can be highly informative to more thoroughly understand how seizures and ASDs are tolerated in the aged rodent brain.

## Conclusions and Future Directions

Considerable understanding of the hyperexcitability and seizures in AD has come from small clinical studies [3] and preclinical work primarily with early-onset AD-associated APP-overexpressing mice [23, 67]. However, the intersection between seizures and AD remains relatively underexplored. Numerous therapeutic targets bridge the intersection between both disorders; additional study is clearly warranted. PSEN2 variants are highly associated with seizures within 5 years of AD diagnosis; this incidence matches that which is associated with APP duplications and is more common than that which is observed with PSEN1 variants [122]. As a result, PSENs, and PSEN2 in particular, should be more frequently studied in isolation to interrogate the effects of PSEN2 variants on seizures in AD. Mice with PSEN2 variants do not demonstrate Aβ plaques [110]. PSEN2 variant models are intriguing to study the pathological intersection between seizures in AD because of the role for PS2 protein that is independent from its γ-secretase activity. In this regard, PSEN2 is an untapped opportunity to define the contributions that Aβ-independent mechanisms may play in the hyperexcitability of AD. PSEN2 manipulation may additionally reveal novel molecular contributors to ictogenesis [123]. There is reason to believe that novel therapies for AD and epilepsy could be bidirectionally uncovered [124]. Basic science has generated a remarkable diversity of preclinical models of AD that have advanced our understanding of its pathophysiological processes. Now, it is incumbent that we further expand the application of these models to comorbid conditions (e.g., epilepsy) to best inform the clinical management and further elucidate the mechanisms of hyperexcitability and its influence on AD trajectory. Prioritizing the studies of seizures in a diversity of AD models will better inform future ASD discovery for elderly patients.
